# Impact of adding a filter for protection from toxic inhalational compounds to the ventilation circuit of mechanically ventilated patients

**DOI:** 10.1186/s40696-016-0015-6

**Published:** 2016-03-01

**Authors:** Eliezer Be’eri, Simon Owen, Mark Shachar, Yaron Barlavie, Arik Eisenkraft

**Affiliations:** 1 Respiratory Rehabilitation Department, Alyn Hospital, Jerusalem, Israel; 2 Versamed Ltd., Tel-Aviv, Israel; 3 Medical Intensive Care Unit, Rambam Medical Center, Haifa, Israel; 4 IDF Medical Corps, Kiryat Ono, Israel; 5 Institute for Research in Military Medicine, The Faculty of Medicine, The Hebrew University of Jerusalem, Jerusalem, Israel; 6 NBC Protection Division, IMOD, HaKirya, 61909 Tel-Aviv, Israel

**Keywords:** Protection, Ventilation, Toxic inhalational compounds, CBRN filter

## Abstract

**Background:**

Standard-issue Chemical-Biological-Radio-Nuclear (CBRN) gasmasks, as used for protection from non-conventional warfare agents or toxic industrial compounds, cannot be used by ventilated patients, leaving them exposed to toxic agents inhaled via their ventilators. This study was conducted to determine the safety of a CBRN filter added to the patient circuit of a ventilator, as a method for affording inhalational protection to ventilated patients.

**Methods:**

A Landrace pig was ventilated sequentially with 3 types of ventilators according to 17 different ventilation protocols, with and without a CBRN filters added in-line to the ventilation tubing for each protocol. For each protocol, physiological parameters, including oxygen saturation, inspired CO_2_, end tidal CO_2_, inspired oxygen, respiratory rate, and pulse rate, as well as airflow parameters including peak inspiratory pressure, positive end expiratory pressure and tidal volume were measured. The impact on the ventilator’s trigger/sensitivity function was evaluated in vitro using a Michigan test lung.

**Results:**

On average, the addition of the CBRN filter resulted in a 16 ml (5 %) decrease (range 0–50 ml) in the tidal volume, a 1.7 cm H_2_O (10 %) decrease (range 1–3 cm H_2_O) in the peak inspiratory pressure, and a 0.1 cm H_2_O (3 %) decrease (range 0–1 cm H_2_O) in the positive end expiratory pressure delivered to the animal. Some ventilators compensated for these airflow changes while others did not, depending on the design of the ventilator’s pressure/flow sensing mechanism. Significant rebreathing occurred when the filter was positioned directly on the animal’s endotracheal tube, but not when positioned on the air outflow port of the ventilator. *In vitro* measurements showed that the addition of the CBRN filter added a mean pressure gradient of 0.45 cm H_2_O to the trigger/sensitivity function of the system.

**Conclusions:**

In-line addition of a CBRN filter to ventilation tubing is a feasible strategy for affording inhalational protection to ventilated patients.

## Background

Dispersion of volatile Toxic Industrial Compounds (TICs) presents a major threat to nearby residents. A scenario of volatile TIC dispersion may occur due to an industrial accident, or as a result of an act of terrorism [1–10]. Protection against TIC exposure can be afforded by wearing a standard issue gasmask, which provides respiratory protection by filtering inspired air through a standard Chemical-Biological-Radio-Nuclear (CBRN) filter that removes toxic agents by adsorbance and absorbance into activated charcoal in the filter [11, 12]. Mechanically ventilated patients, however, are a subgroup for whom standard CBRN gasmasks worn on the face would not provide protection from TICs, because in an environment contaminated with TICs an exposed ventilator would intake contaminated air and transmit it directly to the patient via the ventilation circuit and patient interface (regardless of whether the interface is an endotracheal tube, a tracheostomy cannula or a noninvasive facemask), without flowing through a CBRN filter along the way. A potential vulnerability to TIC exposure thus exists for patients who are ventilated on any machine that intakes atmospheric air. This would include home ventilators that intake air directly from the surrounding environment; ICU or anesthesia ventilators that operate on compressed air from a central air compressor (where the air compressor itself might be exposed to contaminated air); and ventilators that blend compressed air or oxygen (such as from an oxygen cylinder) with ambient air. In addition, for many patients who use ventilatory support only part-time—such as neuromuscular patients on nighttime facemask ventilation—wearing a CBRN gasmask while not on the ventilator might not be a realistic method for TIC protection, because the increased resistance to inspiration caused by the CBRN filter might not be tolerable for patients in chronic respiratory failure, especially at a time of psychological stress and concomitant increased respiratory rate. Although a limited number of ventilators have been custom designed to enable attachment of a CBRN filter to the ventilator’s air intake port for filtering of TICs [13], the majority of home and hospital ventilators currently in use do not have this design feature. To the best of our knowledge, no effective solutions for these ventilated patients in an event of volatile TIC dispersion have been reported in the literature. The current study was therefore aimed at developing a single standardized, universally applicable solution for TIC protection for all mechanically ventilated patients, in both the home and hospital settings, regardless of the type of ventilator being used.

The following possible solutions for ventilated patients were initially considered: attaching a CBRN filter to the air intake port of the ventilator, enclosing the patient and the ventilator in a gas-proof tent, and attaching the ventilator air intake port to a cylinder of compressed air or oxygen, which would be closed to the environment.

Preliminary experiments showed that adapting CBRN filters to cover the air intake ports of ventilators was not a feasible mass solution, because the configurations of air-intake ports differ between different ventilator models, for some of which it was impossible to attach a CBRN filter in a way that would reliably prevent contaminated air from entering. Gas-proof tents are prohibitively expensive, and erecting them is labor-intensive, making them, too, an unrealistic mass solution for all ventilated patients [14]. In addition, use of such tents is not feasible in ICUs, where rapid and unhindered access to the ventilated patient is essential at all times. Nor are such tents suitable for use in chronic-care and rehabilitation facilities, where a single member of staff may be responsible for, and therefore need repeated access to, several patients simultaneously. Cylinders of compressed gas were found to be appropriate for ICU ventilators that operate on compressed air, but not for home ventilators that draw in room air; moreover, this solution would only be effective for a limited period of time, until the contents of the cylinder ran out.

We therefore determined that the only practical, affordable and universally applicable solution to the problem of CBRN protection for ventilated patients during a TIC dispersion incident would be to attach a CBRN filter not to the air-intake port of each ventilator, but to the air-outflow port. This would ensure that even if the ventilator itself was contaminated, all air reaching the patient would be adequately filtered of contaminants; in addition, this solution would ensure that the period of time during which a ventilator could operate during suspected exposure to the toxic compounds would be practically unlimited, and that caregivers would have unhindered access to the ventilated patient at all times. Since the diameter of air-outflow ports on all ventilators conforms to a universal 22 mm standard, mass-production of a CBRN filter that would fit onto the air-outflow port of any model of ventilator in an airtight manner would be both possible and inexpensive.

The solution of attaching a CBRN filter to the air-outflow port of a ventilator, however, raised concerns that the increased resistance to airflow caused by the filter might impact adversely on the efficacy of the patient’s ventilation. For this reason, we elected to evaluate the safety, in an animal model, of adding a CBRN filter to the air-outflow tract of standard mechanical ventilators.

## Methods

### Animal study

**Fig. 1:**
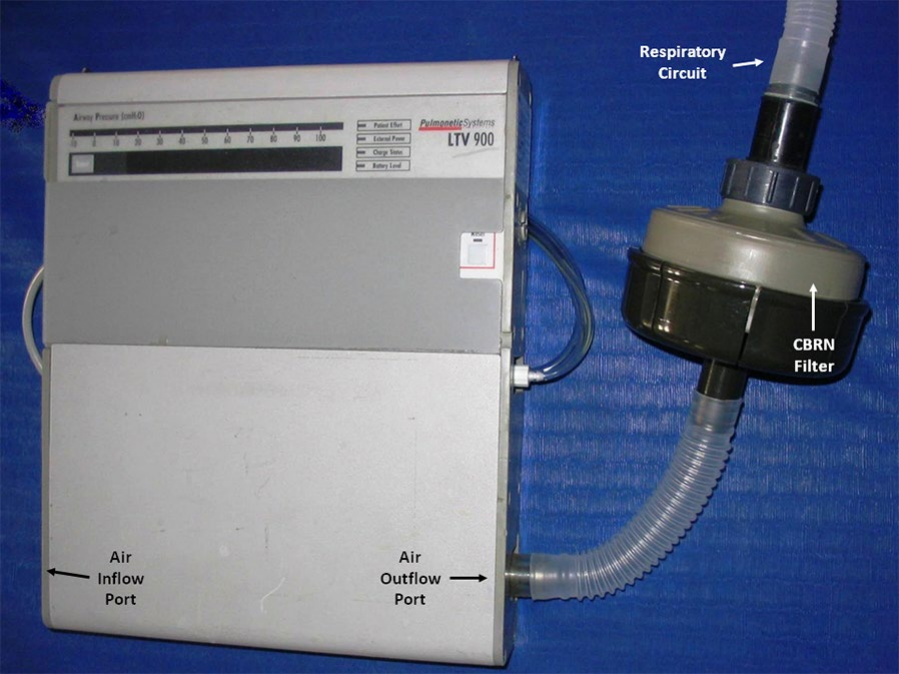
CBRN filter attached to the air-outflow port of a home ventilator

**Fig. 2:**
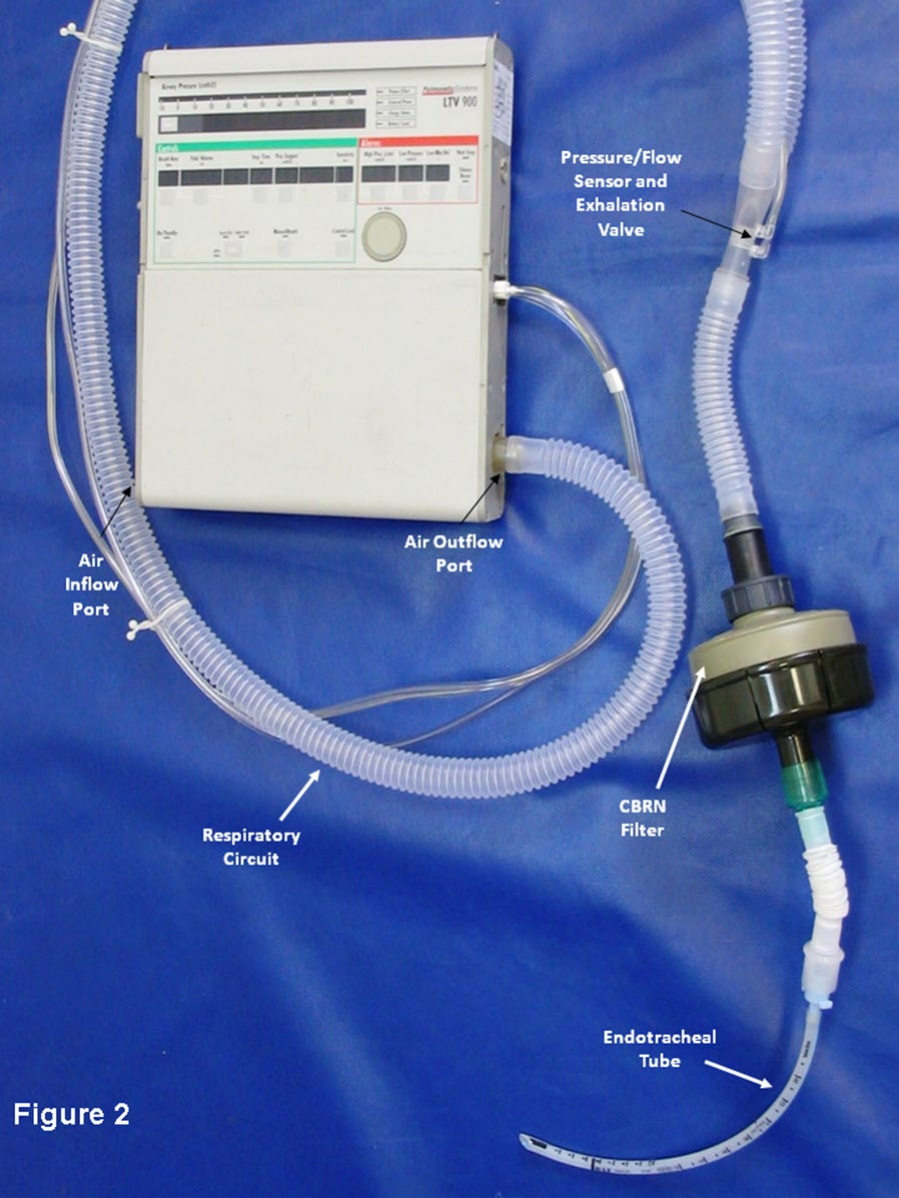
CBRN Filter positioned between an endotracheal tube and the respiratory circuit of a home ventilator

A standard M-80 CBRN filter (Shalon Industries, Kiryat Gat, Israel) was customized to fit standard 22 mm diameter ventilation tubing (Fig. 1). The M-80 filter is commonly issued to both civilians and military personnel in Israel, and is in compliance with generally accepted volume and resistance standards worldwide. A healthy 30 kg Landrace pig was anesthetized with a combination of Diazepam and Ketamine, and intubated with a 7 mm endotracheal tube. The animal was then ventilated using three different models of ventilators representing typical examples of home and ICU ventilators in Israel (Synchrony, Respironics Inc, Murraysville PA; iVent, Versamed Ltd, Israel; and LTV-1000, Pulmonetic Systems, Minneapolis MN), and 17 different ventilation protocols that were designed to be representative of typical home and ICU ventilation settings (Table 1). For each ventilator model and ventilation protocol, the animal was ventilated first without, and then with, a CBRN filter added in-line to the ventilation tubing. Physiological indices (oxygen saturation, inspired CO_2_ [FiCO_2_], end tidal CO_2_ [etCO_2_], inspired oxygen [FiO_2_], respiratory rate [RR], and heart rate [HR]) and airflow parameters (peak inspiratory pressure [PIP], positive end expiratory pressure [PEEP], and tidal volume [TV]) were measured with and without the CBRN filter in place. Protocols 1 through 3 used volume cycled ventilation, protocols 4 through 6 used time-cycled pressure-limited ventilation, and protocols 7 through 12 used flow-cycled (pressure support) ventilation, as typically supplied by bilevel ventilators. For ventilation protocols 1 through 12 the CBRN filter was positioned between the ventilator and the animal’s respiratory circuit (Fig. 1). This was assumed to be the ideal positioning for the CBRN filter, as it achieves CBRN filtration of all inspiratory gas flow, without impacting on expiratory gas flow at all. However, so as to evaluate the potential physiological impact of the CBRN filter when exposed to expiratory airflow, in protocols 13 through 16, using time-cycled pressure-limited ventilation, the CBRN filter was positioned between the animal’s respiratory circuit and endotracheal tube, such that both inspiratory and expiratory gas flow passed through the filter (Fig. 2). Between changes in protocols 1 through 16, and after addition or removal of the CBRN filter, the animal was allowed to stabilize for 3–5 min before physiological and airflow measurements were made. So as to evaluate the potential impact of a longer period of ventilation through an CBRN filter, in protocol number 17 volume-cycled ventilation through a CBRN filter was maintained uninterrupted for 30 min before physiological measurements were recorded.

**Table 1:** Ventilation protocols used

Protocol number	Make of ventilator	Mode	Cycling mechanism	Parameters	Notes
1	Pulmonetic LTV-1000	Assist-control	Volume	TV = 275, PEEP = 4, rate = 20, Ti = 1	a
2	Pulmonetic LTV-1000	Assist-control	Volume	TV = 320, PEEP = 4, rate = 20, Ti = 1	a
3	Pulmonetic LTV-1000	Assist-control	Volume	TV = 400, PEEP = 4, rate = 20, Ti = 1	a
4	Versamed iVent	Assist-control	Time (pressure limited)	PIP = 15, PEEP = 5, rate = 20, Ti = 1	a
5	Versamed iVent	Assist-control	Time (pressure limited)	PIP = 20, PEEP = 5, rate = 20, Ti = 1	a
6	Versamed iVent	Assist-control	Time (pressure limited)	PIP = 25, PEEP = 5, rate = 20, Ti = 1	a
7	Respironics BiPAP Synchrony	Spontaneous-timed	Flow (pressure support)	PIP = 10, PEEP = 5, Ti = 1.4, rate = 20	a
8	Respironics BiPAP Synchrony	Spontaneous-timed	Flow (pressure support)	PIP = 15, PEEP = 5, Ti = 1.4, rate = 20	a
9	Respironics BiPAP Synchrony	Spontaneous-timed	Flow (pressure support)	PIP = 20, PEEP = 5, Ti = 1.4, rate = 20	a
10	Respironics BiPAP Synchrony	Spontaneous-timed	Flow (pressure support)	PIP = 15, PEEP = 4, Ti = 1.4	a
11	Respironics BiPAP Synchrony	Spontaneous-timed	Flow (pressure support)	PIP = 15, PEEP = 6, Ti = 1.4	a
12	Respironics BiPAP Synchrony	Spontaneous-timed	Flow (pressure support)	PIP = 15, PEEP = 8, Ti = 1.4	a
13	Versamed iVent	Assist-control	Time (pressure limited)	PIP = 10, PEEP = 5, rate = 20, Ti = 1	b
14	Versamed iVent	Assist-control	Time (pressure limited)	PIP = 15, PEEP = 5, rate = 20, Ti = 1	b
15	Versamed iVent	Assist-control	Time (pressure limited)	PIP = 20, PEEP = 5, rate = 20, Ti = 1	b
16	Versamed iVent	Assist-control	Time (pressure limited)	PIP = 25, PEEP = 5, rate = 20, Ti = 1	b
17	Versamed iVent	Assist-control	Volume	TV = 260, PEEP = 4, rate = 20, Ti = 1	c

*a* CBRN filter mounted on air outflow port of ventilator

*b* CBRN filter mounted on endotracheal tube

*c* CBRN filter mounted on air outflow port of ventilator, ventilation for 30 min

*Ti* Inspiratory time

Throughout the trial the animal’s state of anesthesia was maintained at a level that prevented the animal from actively opposing mechanical ventilation, and all physiological measurements were made with the animal stable on room air without supplemental oxygen. The trial was approved by the Animal Care Committee of Rambam Medical Center and was conducted at the Animal Research Laboratory of the Technion Medical School/Rambam Medical Center in Haifa, Israel. Animal care and handling were in accordance with the National Institute of Health guidelines for ethical animal research.

### In vitro study

Since the impact of a CBRN filter on a ventilator’s trigger/sensitivity function (i.e., the negative pressure or flow that a patient has to develop spontaneously so as to trigger a mechanical breath from the ventilator) cannot be evaluated on an anesthetized animal that is not breathing spontaneously, this parameter was studied in vitro. First, the resistance versus flow characteristics of a standard-issue CBRN filter were measured empirically using a Windjammer 5.1 inch blower (Amatek Inc. Kent, OH) and an IMT PF300 Flow Analyzer (IMT Medical, Buchs, Switzerland). Then, a Michigan test lung and a Synchrony ventilator set to deliver PEEP at levels of 5, 7 and 9 cm H_2_O were used to evaluate the impact of the CBRN filter on the ventilator’s trigger/sensitivity function. Spontaneous inspiratory effort was simulated by randomly generating brief episodes of negative pressure within the test lung at each of the PEEP values. The negative-pressure waveform that propagated into the ventilation tubing as a result was recorded by two pressure-flow sensors (Y Flow Transducer, Versamed Ltd., Israel) connected in-line between the test lung and the ventilator. The pressure gradient between the sensors was measured first without the CBRN filter in situ, and thereafter with the CBRN filter inserted between the two sensors. The pressure gradient between the pressure-flow sensors was measured using SV Monitor software. The internal volume of the CBRN filter was measured using a water immersion and extraction technique.

## Results

### Animal study

The animal was ventilated for a total of six and a half hours, for approximately half of which the CBRN filter was in situ. Table 2 shows the airflow parameters measured for each ventilation protocol. On average, positioning the CBRN filter in the ventilation circuit resulted in a 16 ml (5 %) decrease in tidal volume (range 0–50 ml), a 1.7 cm H_2_O (10 %) decrease in peak inspiratory pressure (range 1–3 cm H_2_O), and a 0.1 cm H_2_O (3 %) decrease in PEEP across the filter (range 0–1 cm H_2_O). The way that the ventilators responded to the changes caused by addition of the CBRN filter to the ventilation circuit depended on the design of the ventilator’s pressure sensor. The Synchrony machine has an internal pressure/flow sensor located within the ventilator. For this reason, in protocols in which the CBRN filter was positioned on the gas outflow port of the Synchrony ventilator (protocols 7–12), the filter was “downstream” from the ventilator’s pressure/flow sensor. In these instances, the ventilator did not sense or compensate for the pressure/volume drop-off across the filter after the filter was added. The animal consequently received somewhat less ventilation with the filter in situ than without. In contrast, the pressure/flow sensor of iVent and LTV machines is located at the far end of the ventilation tubing, close to the patient. For this reason, the CBRN filter positioned on the gas outflow port of these ventilators (protocols 1–6 and 13–17) was “upstream” from the ventilator’s pressure/flow sensor, enabling the ventilator to sense and compensate for the pressure/volume drop-off caused by the addition of the filter. The animal thus continued to receive the same degree of ventilation even after the addition of the filter.

**Table 2:** Change in air-flow parameters caused by addition of CBRN filter to circuit

Protocol number	Loss of PIP cmH_2_O (%)	Loss of PEEP cmH_2_O (%)	Loss of TV ml (%)	Notes
1	3 (25)	0		a
2	1 (7)	−1 (−20)		a
3	3 (15)	0		a
4	0	0	10 (3)	b
5	1 (5)	0	40 (10)	b
6	3 (12)	0	50 (11)	b
7	1 (10)	0	10 (7)	c
8	1 (7)	0	30 (10)	c
9	2 (10)	1 (20)	20 (5)	c
10	1 (8)	1 (25)	30 (9)	c
11	1 (7)	0	20 (7)	c
12	1 (7)	0	10 (5)	c
13	1 (8)	0	10 (5)	d
14	2 (11)	0	0	d
15	3 (12)	0	−10 (−2)	d
16	3 (10)	1 (20)	−10 (−2)	d
	*Mean* *=* *1.7 (10)*	*Mean* *=* *0.1 (3)*	*Mean* *=* *16.2 (5)*	

*a* Volume cycled ventilation with CBRN filter on air outflow port of ventilator

*b* Time cycled pressure limited ventilation with CBRN filter on air outflow port of ventilator

*c* Flow cycled ventilation with CBRN filter on air outflow port of ventilator

*d* Time cycled pressure limited ventilation with CBRN filter on endotracheal tube

Addition of a filter between the ventilator and the respiratory circuit (protocols 1–12 and protocol 17) did not cause any detectable deterioration in the animal’s respiratory physiology (saturation, etCO_2_, RR and HR remained constant), and there was no evidence of rebreathing (no change in FiO_2_ or FiCO_2_). However, when the CBRN filter was positioned between the respiratory circuit and the endotracheal tube (protocols 13 through 16), evidence of rebreathing emerged within 1 min: FiCO_2_ increased to 1 %, FiO_2_ decreased to 17 %, and oxygen saturation decreased to less than 90 %.

### In vitro study

**Fig. 3:**
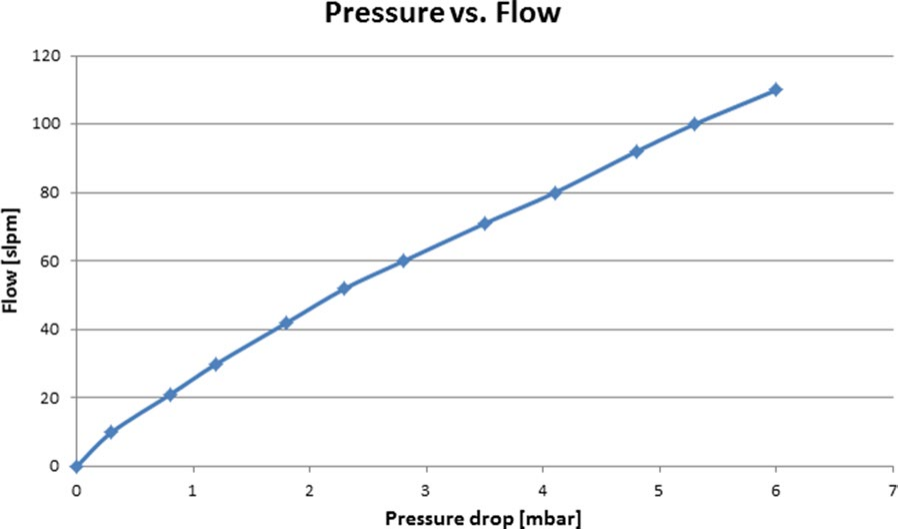
Resistance vs flow of a standard-issue CBRN filter

The resistance versus flow characteristics of the CBRN filter as measured empirically is presented in Fig. 3. Table 3 shows the impact of the CBRN filter on the trigger/sensitivity function of a Synchrony ventilator. The table shows the negative-pressure (“trigger”) gradient generated by the Michigan test lung, as measured by two pressure-flow sensors positioned between the test lung and the ventilator. The negative pressure inspiratory flow generated by each of the simulated inspiratory efforts (i.e. prior to the onset of positive pressure airflow from the ventilator) ranged from 1 to 5 L/min. Without the filter in situ, the intrinsic resistance of the sensors resulted in a mean pressure gradient of 0.14 cm H_2_O between them. After insertion of the filter between the sensors, generation of negative pressure spikes in the test lung showed a mean pressure gradient of 0.59 cm H_2_O (range 0.19–1.39 cm H_2_O) across the filter. Ignoring the intrinsic resistance of the sensors, the filter generated a mean pressure gradient of 0.45 cm H_2_O, a one-third drop-off in trigger pressure. The internal volume of the CBRN filter, representing the dead space added to the respiratory airflow pathway by addition of the filter, was measured as being 140 ml.

**Table 3:** Trigger gradient across the CBRN filter (cm H_2_O)

Pressure generated proximal to filter	Pressure measured distal to filter	Pressure gradient across filter
−0.72	−0.50	0.22
−0.78	−0.43	0.35
−0.86	−0.63	0.23
−1.27	−0.78	0.49
−1.30	−1.11	0.19
−1.53	−1.32	0.21
−1.65	−1.21	0.44
−1.90	−1.36	0.54
−2.37	−1.66	0.71
−2.54	−1.57	0.97
−3.07	−1.68	1.39
−4.04	−2.65	1.39
*Mean: −1.84*	*Mean: −1.24*	*Mean: 0.59*

## Discussion

Manufacturing and chemical plants may pose environmental hazards to nearby residents, due to either accidents or malpractice [1–10]. In addition, in the current era biological and chemical TICs have been used in warfare and acts of terrorism. Ventilated patients in institutions and ambulatory settings may thus be affected by TICs. Nevertheless, current protective protocols do not provide an adequate solution for this population. In this study, the safety of a standard CBRN filter added in-line to the ventilation circuit of an animal model was tested. We show that the addition of a CBRN filter is safe, well tolerated physiologically, and has minimal impact on airflow parameters. It is suggested that this solution may be a useful method for protecting ventilated patients in all settings from TICs.

We are aware of several limitations to this study. Technical constraints prevented us from evaluating the loss of tidal volume across the CBRN filter under conditions of volume-cycled ventilation. Changes in this parameter, however, are unlikely to differ from those found under conditions of time-cycled and flow-cycled ventilation. In addition, inspiratory time was not measured, as this parameter was fixed for all the ventilation protocols used in the trial and thus would not change with the addition of a CBRN filter to the ventilation circuit.

Application of this technique on a mass scale may require several precautions in order to minimize the risk of harm being caused to the patient by the filter. It is important to ensure that the CBRN filter is positioned directly on the air outflow port of the ventilator, and not on the patient’s tracheostomy or endotracheal tube, as this will lead to dangerous rebreathing of exhaled CO_2_, most likely due to accumulation of CO_2_ in the dead space of the CBRN filter. Although off-gassing (i.e. second-hand exposure to toxins in air exhaled from a contaminated patient) is a concern for caregivers treating patients exposed to nerve agents, and positioning the CBRN filter directly on the patient’s endotracheal tube might provide some protection against this, for most TICs this is not a real concern, and the danger to the patient of hypercapnia from rebreathing far outweighs the theoretical benefit of minimizing off-gassing by placing the CBRN filter directly on the endotracheal tube.

For ventilators with an externally located pressure/flow sensor, such as most volume-cycled and time-cycled ventilators, no readjustment of tidal volume or PIP should be necessary after addition of a CBRN filter to the ventilation circuit, provided the CBRN filter is correctly positioned on the ventilator outflow port, “upstream” from the pressure/flow sensor. However, for ventilators with an internally located pressure/flow sensor, such as bi-level ventilators, it may be advisable to increase the inspiratory positive airway pressure (IPAP) by 2 cm H_2_O so as to compensate for potential pressure drop-off after addition of a CBRN filter. Although some patients on home ventilation will be able to perform such an adjustment competently and safely, and will remember to return the ventilator settings to their baseline after removal of the CBRN filter, the potential for error on the part of less aware home ventilated patients is real, particularly during a time of high stress and anxiety. For this reason, public health authorities should consider whether or not adequate education of this population is feasible before issuing a general recommendation to change the parameters of bi-level ventilators when attaching a CBRN filter. It may be more prudent to recommend that healthcare workers make such changes, wherever possible, while advising patients not to adjust their ventilator settings themselves.

Patients breathing spontaneously should be advised that the addition of the filter may cause a sensation of increased effort of breathing, due to the increase in resistance to trigger flow caused by the CBRN filter. This may necessitate increasing the mandatory number of breaths delivered by the ventilator per minute as a means of compensation. In addition, the sensation of increased resistance to initiating a breath that is caused by the addition of a CBRN filter may be mitigated in part by increasing the patient’s PEEP setting or adjusting the sensitivity of the ventilator’s trigger function.

Both public health authorities and patients should be informed as to the recommended duration of use of a CBRN filter once it has been unsealed and inserted into the patient’s ventilation circuit. When exposed to the TIC ambient air concentrations that are typically encountered in industrial accidents or acts of terror, CBRN filters provide adequate protection for a minimum of 8 h, at all clinically used ventilation minute volumes. There is therefore no need to replace the filter prior to that, and it is highly unlikely that a single TIC exposure incident would last longer than that timeframe. However, in the event of prolonged exposure beyond 8 h, a new filter should be inserted as soon as is feasible thereafter. If a new filter has been opened but not exposed to active airflow, it can be resealed and stored for up to 12 months without loss of efficacy. Although addition of a CBRN filter to a patient’s ventilation circuit protects the patient from inhaling TICs, the patient remains exposed to these agents through his mucous membranes. For this reason, patients being ventilated via a tracheostomy should wear a standard gasmask to protect the mucous membranes of their eyes, nose and mouth, in addition to adding a CBRN filter to their ventilation tubing. This may not be feasible, however, for patients being ventilated via an endotracheal tube or facemask.

Another concern is that of a possible exothermic reaction between the charcoal of the CBRN filter and enriched oxygen, which is commonly used by ventilated patients. This was tested separately in a study that showed no such reaction, and that oxygen flow through a CBRN filter is safe [15].

## Conclusions

In-line addition of a CBRN filter to the ventilation circuit represents a feasible strategy for affording inhalational protection from toxic inhalational compounds to the general population of home and ICU ventilated patients. Further clinical studies are necessary to confirm these findings.

## Abbreviations

CBRN: Chemical-Biological-Radio-Nuclear
TICs: toxic industrial compounds
PEEP: positive end expiratory pressure
PIP: peak inspiratory pressure
TV: tidal volume
FiCO_2_: inspired CO_2_
etCO_2_: end tidal CO_2_
FiO_2_: inspired oxygen
RR: respiratory rate
HR: heart rate
IPAP: inspiratory positive airway pressure
Ti: inspiratory time
